# Dr. Kinglake's Notice

**Published:** 1805-07-01

**Authors:** Robert Kinglake

**Affiliations:** Taunton


### *To the Editors of the Medical and Physical Journal.*

Gentlemen,

In your Review* of my reply to Mr. Edlin's two cases of gout, occurs the following remark. "In some concluding observations the author gives a case, in which the hot treatment of gout proved fatal. Though we have no doubt of the truth of every thing here stated, yet we wish, for the sake of the incredulous part of the public, that the name at least of the consulting physician had been added. We are aware that this cannot always be done, from motives of delicacy to the surviving family of the deceased; but we regret the want of it exceedingly in this case." The omission here regretted, was occasioned by the physician alluded to, being dead; the mentioning of which circumstance, it was thought, would tend to draw undue suspicion on the credibility of the case. However, as the suppression of the name is lamented, it behoves me to disclose it. The physician referred to was the late Dr. Vivian of Oxford. The widow, and several children of the deceased patient, are still living, and shall likewise be made known to any person who would wish their attestation of the facts stated in my detail of the case. I am, &c.

*Taunton, May 20, 1805.* ROBERT KINGLAKE.

---

* See Medical and Physical Journal, No. 75, p. 464.

